# A Balance of BMP and Notch Activity Regulates Neurogenesis and Olfactory Nerve Formation

**DOI:** 10.1371/journal.pone.0017379

**Published:** 2011-02-23

**Authors:** Esther Maier, Hanna Nord, Jonas von Hofsten, Lena Gunhaga

**Affiliations:** Umeå Center for Molecular Medicine, Umeå University, Umeå, Sweden; Stockholm University, Sweden

## Abstract

Although the function of the adult olfactory system has been thoroughly studied, the molecular mechanisms regulating the initial formation of the olfactory nerve, the first cranial nerve, remain poorly defined. Here, we provide evidence that both modulated Notch and bone morphogenetic protein (BMP) signaling affect the generation of neurons in the olfactory epithelium and reduce the number of migratory neurons, so called epithelioid cells. We show that this reduction of epithelial and migratory neurons is followed by a subsequent failure or complete absence of olfactory nerve formation. These data provide new insights into the early generation of neurons in the olfactory epithelium and the initial formation of the olfactory nerve tract. Our results present a novel mechanism in which BMP signals negatively affect Notch activity in a dominant manner in the olfactory epithelium, thereby regulating neurogenesis and explain why a balance of BMP and Notch activity is critical for the generation of neurons and proper development of the olfactory nerve.

## Introduction

The sense of smell among animals is used for food search and evaluation, localization of predators or preys, and for chemical detection of individuals. Olfactory sensory neurons in the main olfactory sensory epithelium transduce odour signals via the olfactory nerve to the olfactory bulb, from where the sensory input is transmitted to the olfactory cortex to be processed. During embryonic development, both the sensory epithelium and the olfactory nerve arise from the olfactory placode [1]. The mature olfactory nerve is composed of axons originating from post-mitotic olfactory sensory neurons in the epithelium of the nasal cavity. However, the molecular mechanisms that regulate the initial formation of the olfactory tract remain poorly defined.

Olfactory placodal cells are specified already at late gastrula stages in chick [2], but it is not until Hamburger and Hamilton stage 14 (∼22 somites) [3], that the olfactory placode becomes morphologically visible as an epithelial thickening of the head ectoderm near the telencephalon. Coincident with the invagination of the placode into the olfactory pit, the majority of the earliest forming neurons migrate away from the olfactory epithelium, and are often referred to as epithelioid cells or migratory mass [1], [4], [5], [6], [7]. The function of these early-delaminating neurons remains unclear, although they have been suggested to perforate the olfactory epithelial basal lamina creating openings later utilized by emerging olfactory axons [4], and to build up the olfactory nerve tract [5], [7]. Shortly thereafter, olfactory sensory neurons situated in the epithelium extend their axons, and around stage 20 in chick, the olfactory nerve can be detected [4]. Despite the morphological characterisation of early events in olfactory nerve formation, little is known about how extrinsic signals control the early events of neurogenesis and the generation of the first migratory neurons from the olfactory sensory epithelium.

Notch and Bone morphogenetic protein (BMP) signals are known to affect cell differentiation. BMP signals have been suggested to play a role during neurogenesis in the olfactory sensory epithelium [8], [9], [10], and several members of the Notch signaling pathway are expressed in the olfactory epithelium of mouse [11], [12]. The Notch receptors are activated by Delta and Serrate/Jagged ligands [13], which promote proteolytic cleavage and the release of the Notch intracellular domain (ICD). The activated Notch-ICD translocates into the nucleus and interacts with the transcription factor CSL/RBP-Jκ and the co-activator Mastermind (MAM) to activate target genes such as *Hes1* and *Hes5* (reviewed in [14], [15]), and *Hes* expression can therefore be used as a read out for Notch activity [16], [17]. However, whether and how Notch and/or BMP signals regulate the generation and migration of migratory neurons and the initial formation of the olfactory nerve has not been resolved.

Here we have addressed these issues, by manipulating Notch or BMP signals in chick explant and whole embryo assays, and we conclude that these pathways play different roles during the generation of early migratory neurons leaving the olfactory epithelium. By using optical projection tomography (OPT), a 3D imaging technique, we provide evidence that Notch and BMP signaling are important for the initial formation of the olfactory nerve. Our results indicate that Notch activity is required for the maintenance of a progenitor pool of cells in the olfactory placode, and that over-activation of Notch signals in olfactory placodal cells abolishes or strongly reduces the thickness of the olfactory nerve. Finally, our results provide evidence that BMP signals negatively regulate Notch activity in olfactory placodal cells, indicating that a balance of BMP and Notch activity is critical for proper development of the olfactory nerve.

## Results

### Modulated BMP or Notch activity disturbs olfactory nerve formation

To examine the effects of BMP and Notch signaling on early neurogenesis and the initial formation of the olfactory nerve, we electroporated stage 12/13 chick embryos, prior to the onset of neurogenesis [6], in the olfactory placodal region to transfer a control *green fluorescent protein* (*GFP*) vector alone or together with the following constructs; I) a dominant negative (dn) *MAMLI* vector, that acts as an inhibitor of Notch signaling [18], II) a ca*Notch1-ICD* (ca*Notch1*) vector containing a constitutively active Notch receptor [19], III) a *Noggin* vector, which act as a BMP inhibitor [20], IV) a constitutively active BMP receptor, *Alk6* (*Bmpr1b*), vector [21]. The chick embryos were cultured *in ovo* to approximately stage 27. Embryos with GFP expression within the olfactory region were selected for further analyses, stained with a Tuj1 antibody to visualise the nerve tracts [22] and assayed using a whole embryo 3D-OPT imaging technique.

**Figure 1:**
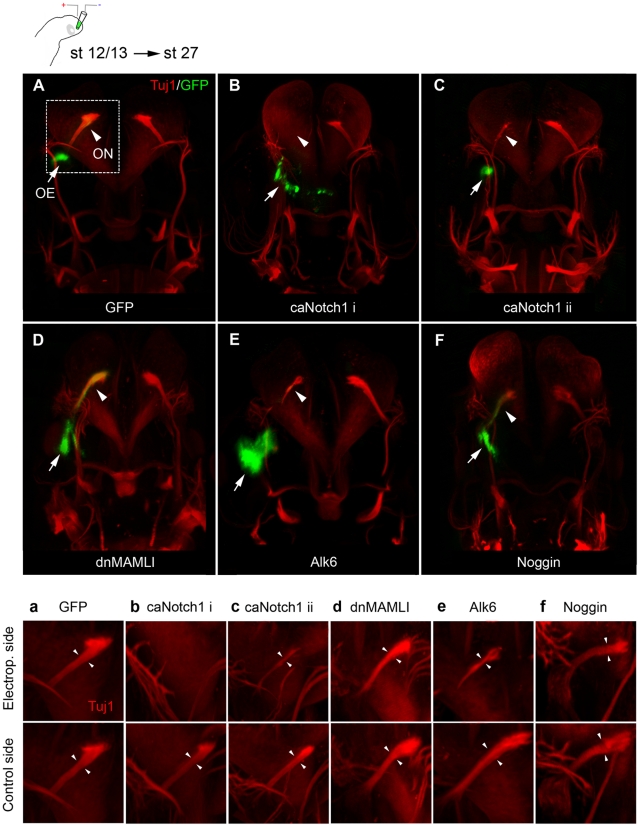
The formation of the olfactory nerve is depending on Notch and BMP activity. (**A–F**, **a–f**) Tuj1^+^ neurons (red) were detected using whole embryo 3D-OPT imaging of chick embryo heads at stage 27, after *in ovo* electroporation in the olfactory placode at stage 12/13 using a control *GFP*-construct (green) alone or together with constructs modulating Notch or BMP activity. (**A**,**a**) The olfactory nerve remains unaffected by electroporation of *GFP* as compared to the non-electroporated control side. (**B**,**b**,**C**,**c**) A ca*Notch1* construct co-electroporated with *GFP* resulted in a total loss of (B,b,:caNotch1 i) or a severely reduced olfactory nerve (C,c,: caNotch ii). (**D**,**d**) Inhibition of Notch signaling by electroporation of a dn*MAMLI* construct did not significantly disturb the generation of the olfactory nerve. (**E**,**e**) Over-activation of BMP activity by electroporating an *Alk6* vector, resulted in an olfactory nerve with reduced thickness. (**F**,**f**) *In ovo* electroporation of a *Noggin* vector resulted in an olfactory nerve with slightly reduced thickness. Arrowheads indicate the olfactory nerve (ON), arrows indicates the olfactory epihelium (OE).

All control-electroporated embryos (n = 6) exhibited normal morphology of the olfactory pit and the olfactory nerve (Fig. 1A,a and Movie S1) and also in all dn*MAMLI*-transfected embryos (n = 6), where Notch signaling was blocked, the olfactory nerve appeared to be unaffected (Fig. 1D,d). In contrast, in all ca*Notch1*-electroporated embryos (n = 7), the olfactory nerve was either abolished (4/7), classified as caNotch1 i (Fig. 1B,b and Movie S2) or reduced in thickness (3/7), classified as caNotch1 ii (Fig. 1C,c). Also in all *Alk6*-electroporated embryos (n = 8), where BMP signaling was elevated, the olfactory nerve was reduced in thickness (Fig. 1E,e), and a similar phenotype was observed in *Noggin*-transfected embryos (3/5) (Fig. 1F,f).

To determine whether increased cell death accounted for the absence of the olfactory nerve or reduction of nerve thickness, we analyzed the expression of the cell death marker activated (a) Caspase3. There was no change in cell death in ca*Notch1*-, *Alk6*- or *Noggin*-electroporated embryos compared with control *GFP*-electroporated embryos (Fig. S1; Methods S1). Thus, both modulated Notch and BMP activity disrupts the formation of the olfactory nerve, without significantly affecting cell death. Moreover, since we never detected any misrouted axons or stray Tuj1 neurons in the analysed embryos (Fig. 1b–f) it is not likely that the reduction in nerve thickness or complete absence of the olfactory nerve is caused by disturbed migration of the migratory neurons or that later axonal path finding is perturbed. We therefore focused our further studies on the effects BMP and Notch activity exert on neuron numbers within the olfactory epithelium and the migratory mass.

### The migratory epithelioid cells are of post-mitotic neuronal character

**Figure 2:**
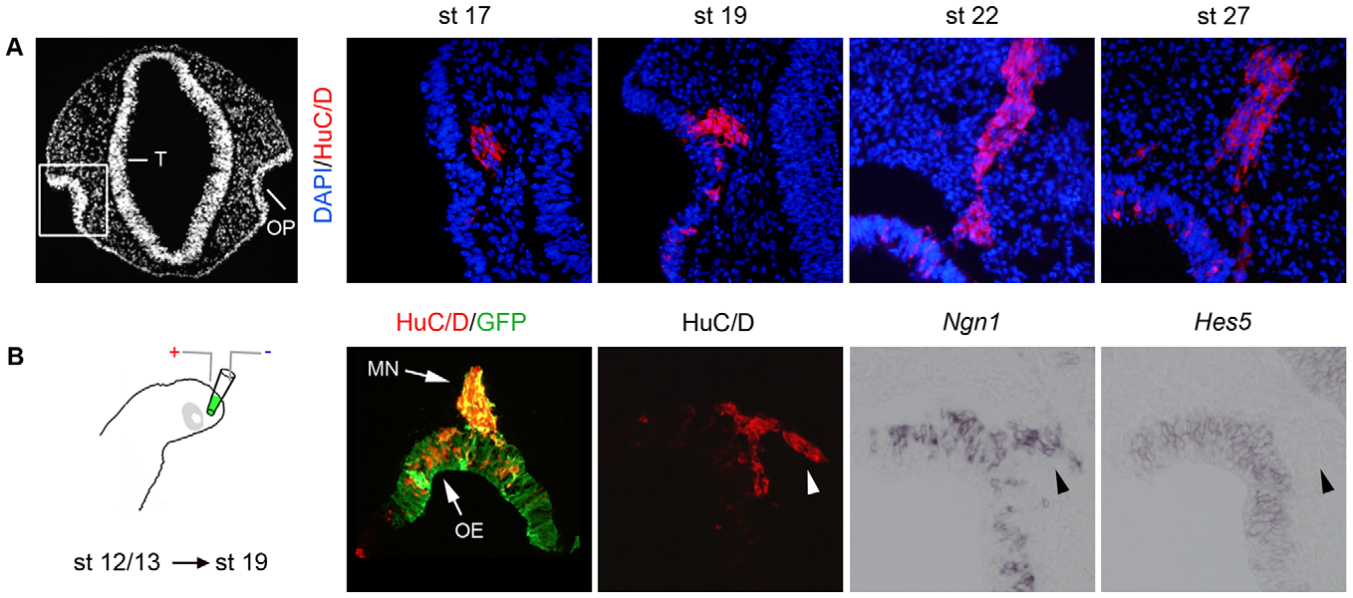
The migratory epithelioid cells are of post-mitotic neuronal character. (**A**) A DAPI transversal section of a stage 19 forebrain, showing the location of the olfactory pit (OP) in correlation to the telencephalon (T). At stage 17, 19, 22 and 27 HuC/D expression is detected in migratory cells emanating from the olfactory epithelium. (**B**) Stage 12/13 chick embryos were electroporated *in ovo* in the olfactory placodal region using a construct encoding GFP, and cultured to approximately stage 19. Embryos with GFP expression in the olfactory pit region were selected, sectioned and analysed. All GFP^+^ migratory cells express HuC/D (MN), and the migratory HuC/D^+^ neurons express *Ngn1*, but not *Hes5* (arrowheads). Expression of HuC/D, *Ngn1* and *Hes5* can be detected in the olfactory epithelium (OE).

First we characterised the migratory cell population leaving the olfactory epithelium by sectioning stage 17, 19, 22 and 27 chick embryos and analyzed the expression of HuC/D, a post-mitotic neural marker [5]. At all stages examined all of the migratory cells appear to express HuC/D and were observed in a cluster extending from the olfactory epithelium to the forebrain (Fig. 2A). To verify that all early migratory cells exhibited a neuronal character, we electroporated stage 12/13 chick embryos *in ovo* in the olfactory placodal region, and cultured the embryos to stage 19 (Fig. 2B). Embryos with GFP expression in the olfactory pit region were selected for further analyses and stained with an HuC/D antibody. In all embryos examined, although GFP expressing cells were of both HuC/D^−^ and HuC/D^+^ character in the olfactory epithelium, all migratory GFP^+^ cells expressed HuC/D and were located in a restricted cluster (Fig. 2B). In addition, migratory HuC/D^+^ neurons also expressed *Ngn1*, a known neuronal marker [23], but not *Hes5*, which defines progenitor cells [24] (Fig. 2B). Thus, the migratory cells leaving the olfactory epithelium are post-mitotic neurons and migrate as a restricted group of cells towards the forebrain.

### Both elevation and inhibition of BMP activity reduce the number of migratory neurons

**Figure 3:**
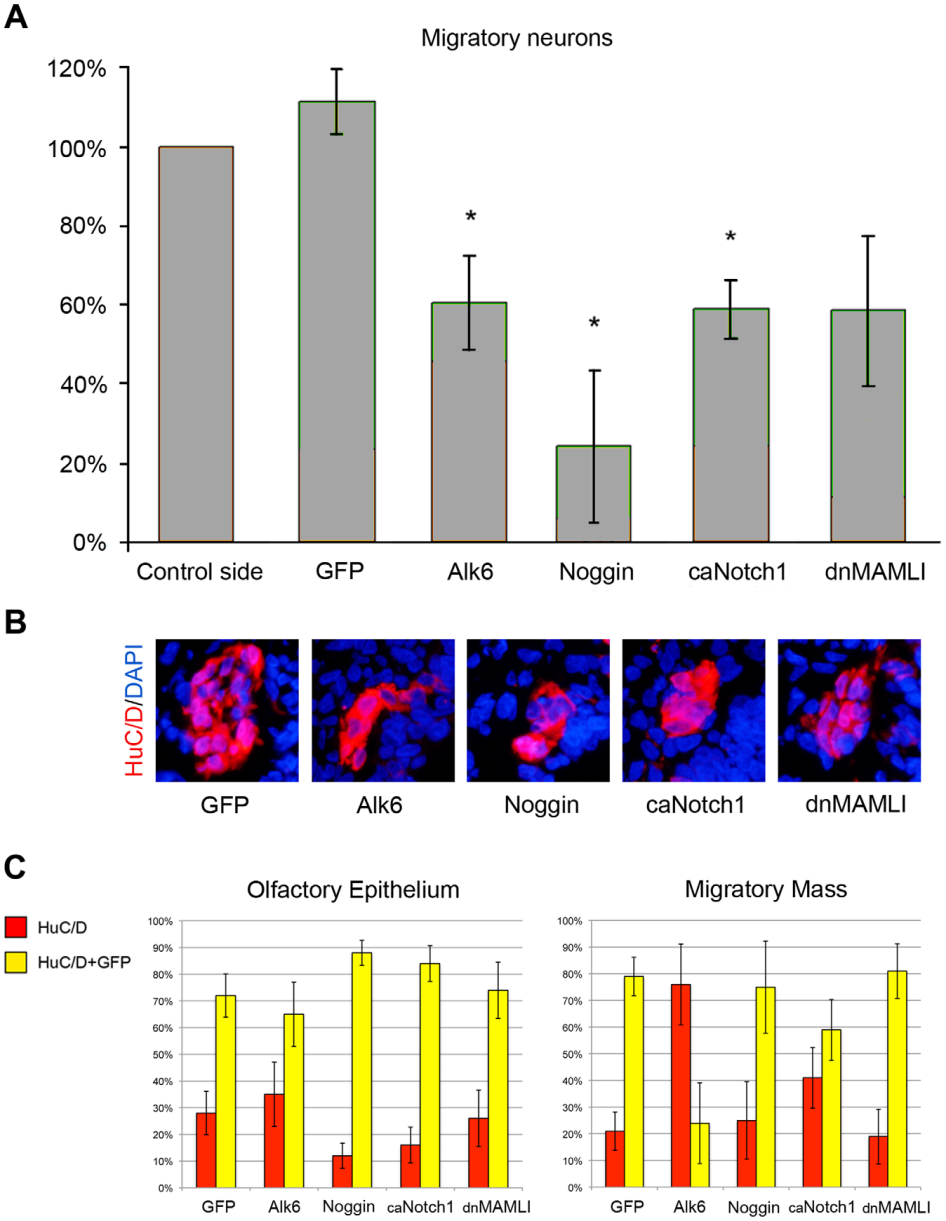
The generation of migratory neurons is regulated by Notch and BMP signaling. (**A**) The total number of migratory neurons was unaffected by electroporation of *GFP*, but was significantly decreased (* = p<0,05 by *t-*test) in the *Alk6*-, *Noggin*- and ca*Notch1*-electroporated olfactory pit compared to the non-electroporated side. Electroporation of dn*MAMLI* resulted in a non-significant decrease of migratory neurons compared to the non-electroporated side. (**B**) Immunohistochemical images of the generation of HuC/D^+^ migratory neurons in electroporated embryos. Both gain-and-loss of BMP and Notch function reduced the number of migratory HuC/D^+^ neurons. (**C**) Graphs indicate the percentages of HuC/D^+^ neurons (red) and HuC/D^+^/GFP^+^ neurons (yellow) in the olfactory epithelium and in the migratory mass. Error bars in A and C indicate s.e.m.

BMP activity has previously been shown to play an important role in the early specification and patterning of the olfactory placode [2], [8], and for the differentiation of neurons in the olfactory sensory epithelium [8], [9], [10]. To examine in more detail how BMP signals affect the development of migratory neurons from the olfactory epithelium in intact embryos, we electroporated *GFP* alone or together with *Alk6* or *Noggin* in stage 12/13 chick embryos in the olfactory placodal region, and cultured the embryos *in ovo* to approximately stage 19. Embryos with GFP expression in the olfactory pit region were selected for further analyses, and differentiated neurons were detected by HuC/D antibody staining. To evaluate whether BMP activity affects the generation of migratory neurons, we quantified the total number of migratory neurons in the electroporated embryos. In *Alk6*-transfected embryos (n = 7), where BMP signaling was elevated, the total number of migratory neurons was significantly decreased compared to the non-electroporated control side (Fig. 3A,B). Also in *Noggin*-electroporated embryos (n = 7), the total number of migratory neurons was significantly decreased compared to the non-electroporated control side (Fig. 3A,B). Thus, both gain and loss of BMP activity reduce the number of migratory neurons, indicating that the generation of migratory neurons is dependent on BMP levels in the olfactory epithelium.

Next we examined whether the decrease of migratory neurons was due to reduced generation of neurons in the olfactory epithelium, or due to a disturbed ability of the neurons to leave the epithelium. To evaluate this issue, we quantified neurons situated in the olfactory epithelium and in the migratory mass, respectively and distinguished between electroporated (GFP^+^) and non-electroporated (GFP^−^) HuC/D positive neurons. We analysed the percentage of electroporated and non-electroporated neurons located in the epithelium and migratory mass (Fig. 3C), based from raw data presenting the total numbers of electroporated and non-electroporated neurons in both regions (Fig. S2). In *Alk6*-transfected embryos (n = 7), the percentage of HuC/D^+^/GFP^+^ neurons was decreased at a larger extent in the migratory mass than in the olfactory epithelium, compared to *GFP*-electroporated embryos (n = 6) (Fig. 3C). Thus, elevated BMP signals in the olfactory epithelium appear to suppress neurons ability to leave the epithelium, and thereby reduce the number of migratory neurons. In *Noggin*-electroporated embryos (n = 7), the percentage of HuC/D^+^/GFP^+^ neurons in the olfactory epithelium was increased compared to *GFP*-electroporated embryos (n = 6) (Fig. 3C), whereas the percentage of HuC/D^+^/GFP^+^ neurons in the migratory mass was almost unaffected (Fig. 3C). Thus, inhibition of BMP activity reduces the differentiation of neurons, but does not affect the ability of the neurons to leave the olfactory epithelium.

### Inhibition of Notch activity diminishes the proliferative pool of cells

We next turned to the issue of how Notch signals regulate the generation of olfactory neurons. *Notch1*, *Delta1*, *Serrate1* and *Serrate2*, members of the Notch family, are expressed in the olfactory placode and olfactory pit (Fig. S3A,B), where the ligand *Delta1* is expressed both earlier and stronger compared to *Serrate1* and *Serrate2* (Fig. S3A,B). First, we addressed how Notch signals regulate the generation of olfactory neurons in intact chick embryos, by electroporating GFP alone or together with dn*MAMLI* or ca*Notch1* in stage 12/13 chick embryos in the olfactory placodal region, and cultured the embryos *in ovo* to approximately stage 19. In ca*Notch1*-electroporated embryos (n = 7), the total number of migratory neurons was significantly reduced compared to the non-electroporated control side (Fig. 3A,B). Furthermore, we observed a shift of HuC/D^+^/GFP^+^ neurons from the migratory mass to the olfactory epithelium compared to *GFP*-electroporated control embryos (n = 6) (Fig. 3C). Thus, activation of Notch signaling negatively controls the generation of migratory neurons, and also suppress neurons ability to leave the epithelium.

Surprisingly, dn*MAMLI*-electroporated embryos (n = 7) generated less migratory neurons compared to the non-electroporated control side (Fig. 3A,B). Moreover, these embryos did not exhibit any change in percentage of HuC/D^+^/GFP^+^ neurons in the migratory mass compared to control *GFP*-electroporated embryos (n = 6) (Fig. 3C), indicating that the ability of neurons to leave the epithelium was unaffected. A conceivable explanation is that inhibition of Notch activity at stage 12/13, prior to the onset of neurogenesis in the olfactory epithelium, diminished the proliferating pool of cells. Implicating that an increase in differentiation from a smaller progenitor pool leads to a similar percentage of neurons in the olfactory epithelium and the migratory mass as control embryos exhibit. To examine this issue, we analysed the number of proliferating cells in dn*MAMLI*- and control *GFP*-electroporated embryos by using the cell proliferation marker pHistone3. In dn*MAMLI*-electroporated embryos there was a significant decrease in cell proliferation compared with control embryos (Fig. S4; Methods S1). Thus, prior to the onset of neurogenesis, inhibition of Notch activity appears to diminish the proliferative pool of cells within the olfactory epithelium.

### Notch signals initially affect the *Hes5* positive cells of the olfactory neuron lineage

**Figure 4:**
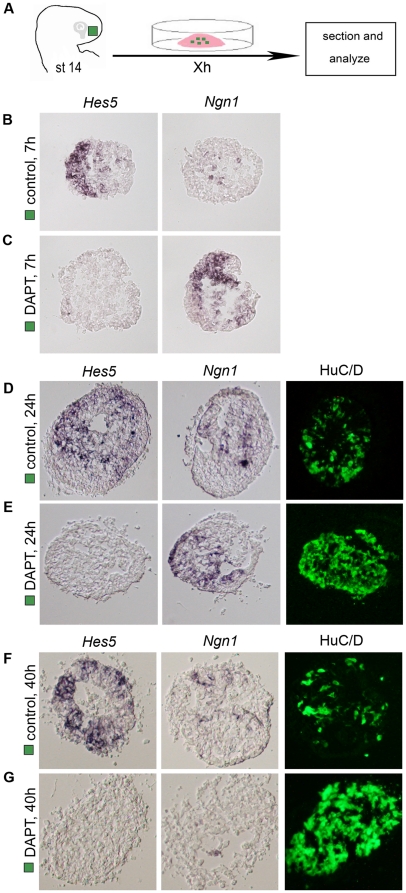
Notch activity is required to maintain *Hes5* positive progenitor cells. (**A**) Schematic drawing depicting the explant method. Stage 14 olfactory placodal (OP) explants (green box) were isolated, separated from the head mesenchyme and cultured *in vitro* for different time points. (**B**) OP explants cultured for 7 h generated *Hes5* ^+^ cells, but no or only a few *Ngn1* ^+^ cells were detected (n = 12). (**C**) OP explants cultured for 7 h in the presence of DAPT (50 µM) generated many *Ngn1* ^+^ precursors, but no *Hes5* ^+^ progenitor cells (n = 12). (**D**, **F**) OP explants cultured for 24 (n = 20) and 40 h (n = 12) generated *Hes5* ^+^, *Ngn1* ^+^ and HuC/D^+^ cells throughout the explants. (**E**) OP explants cultured for 24 h in the presence of DAPT (50 µM) generated many *Ngn1* ^+^ cells and HuC/D^+^ neurons, but no *Hes5* ^+^ progenitor cells (n = 20). (**G**) OP explants cultured for 40 h in the presence of DAPT (50 µM) generated many HuC/D^+^ neurons, but no *Hes5* ^+^ or *Ngn1* ^+^ cells (n = 12).

Neurogenesis in the olfactory epithelium occurs in an ordered manner with defined cell populations, such as progenitor cells, immediate neuronal precursors and post-mitotic neurons. To elucidate in more detail which cell population in the olfactory neuron lineage is initially affected by the loss of Notch activity, we established an explant assay of olfactory epithelial cell differentiation. Olfactory placodal (OP) explants of stage 14 chick embryos were cultured for different time points (Fig. 4A), alone or together with DAPT, an inhibitor of the γ-secretase enzyme, that catalyses the cleavage of the intracellular domain of Notch after ligand binding [25]. The underlying head mesenchyme was removed from the OP explants to avoid indirect effects from these cells. After culture, we analysed the expression of *Hes5*, *Ngn1* and HuC/D.

OP explants cultured alone for 7 h generated mostly *Hes5* ^+^ progenitor cells, and only a few *Ngn1* ^+^ cells throughout the explants (Fig. 4B), whereas in the presence of DAPT (50 µM), the generation of *Hes5* ^+^ cells was blocked and the expression of *Ngn1* ^+^ neuronal precursors was up-regulated (Fig. 4C). OP explants cultured alone for 24 h generated *Hes5* ^+^, *Ngn1* ^+^ and HuC/D^+^ cells (Fig. 4D), but in the presence of DAPT (50 µM) the generation of *Hes5* ^+^ progenitor cells was blocked and the expression of *Ngn1* ^+^ neuronal precursors and HuC/D^+^ neurons was up-regulated (Fig. 4E). After 40 h of culture OP explants cultured alone still generated *Hes5* ^+^, *Ngn1* ^+^ and HuC/D^+^ cells throughout the explants (Fig. 4F). In contrast, in OP explants cultured in the presence of DAPT (50 µM), the generation of *Hes5* ^+^ and *Ngn1* ^+^ cells was blocked and most cells expressed HuC/D (Fig. 4G), characteristic of post-mitotic neurons. Taken together, Notch activity maintains prospective olfactory epithelial cells in a progenitor state, and in the absence of Notch signaling, *Hes5* ^+^-progenitor cells differentiate prematurely in a synchronized manner into post-mitotic neurons.

To verify these findings *in vivo*, we electroporated stage 12/13 chick embryos and cultured to approximately stage 19. In all dn*MAMLI*-electroporated embryos (n = 5) a decrease of *Hes5* expression was detected in the olfactory epithelium, and an increase in *Ngn1* and HuC/D expression in the epithelium and migratory mass was observed (Fig. S5). Thus, also *in vivo* in chick, inhibition of Notch signals diminishes the *Hes5* expressing progenitor cell pool in the olfactory epithelium.

### BMP signals negatively regulate Notch activity in the olfactory epithelium

**Figure 5:**
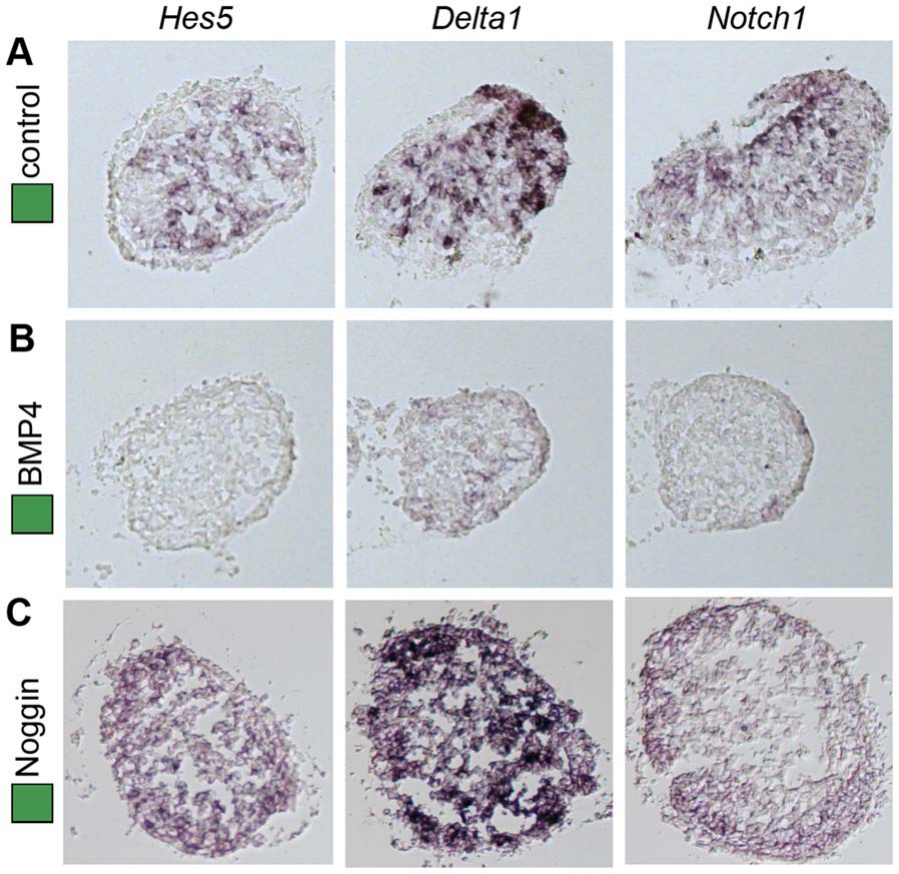
BMP signals negatively regulate Notch activity in olfactory placodal cells. (**A**) OP explants (n = 24) cultured alone generated *Hes5* ^+^, *Delta1* ^+^and *Notch1* ^+^ cells in a scattered manner. (**B**) In OP explants cultured together with BMP4 (35 ng/ml) (n = 12), the generation of *Hes5* ^+^, *Delta1* ^+^and *Notch1* ^+^ cells was blocked. (**C**) In OP explants cultured together with Noggin (n = 18), the generation of *Hes5* ^+^ and *Delta1* ^+^ cells was increased, while the generation of *Notch1* ^+^ cells was unaffected.

Since modulations of either the Notch or the BMP signaling pathway affected the generation of migratory neurons and caused disturbed formation of the olfactory nerve, we examined how these signaling pathways interact in the olfactory epithelium. To analyse this we cultured stage 14 OP explants alone or together with BMP4 (5 nM) or Noggin. OP explants were cultured for 24 h, corresponding in time to approximately stage 20 embryos, and analyzed by *in situ* hybridization on consecutive cryo-sections for the expression of *Notch1*, *Delta1* and *Hes5*. OP explants cultured alone expressed *Notch1*, *Delta1* and *Hes5* in a salt-and-pepper pattern (Fig. 5A). In OP explants cultured together with BMP4 (35 ng/ml), the generation of *Notch1* ^+^, *Delta1* ^+^ and *Hes5* ^+^ cells was inhibited (Fig. 5B). In contrast, in OP explants cultured in the presence of Noggin, *Delta1* and *Hes5* expression was up-regulated throughout the explants, whereas the generation of *Notch1* ^+^ cells was not apparently affected (Fig. 5C). Thus, elevated BMP signals negatively regulate Notch activity in the olfactory epithelium.

**Figure 6:**
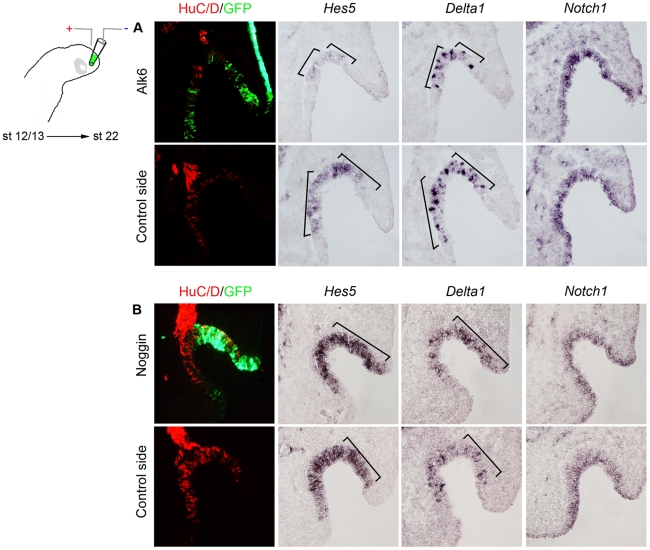
BMP signals negatively regulate Notch activity *in vivo*. (**A**, **B**) *In ovo* electroporation of stage 12/13 in the olfactory placodal region using a GFP construct together with a *Alk6* construct or a *Noggin* construct and cultured to stage 22. (**A**) In *Alk6*-electroporated embryos the expression domain of *Hes5* and *Delta1* decreased, while the generation of *Notch1* ^+^ cells was unaffected. (**B**) In *Noggin*-electroporated embryos the expression domain of *Hes5* and *Delta1* expanded, while the generation of *Notch1* ^+^ cells was unaffected.

To further strengthen these findings, we modulated BMP activity in the olfactory placode by electroporating stage 12/13 chick embryos, cultured to approximately stage 22 and monitored the expression of *Notch1*, *Delta1* and *Hes5*. All control *GFP*-electroporated embryos (n = 4) exhibited a normal expression pattern of *Notch1*, *Delta1* and *Hes5* (data not shown). In all *Alk6* electroporated embryos (n = 5), where BMP signaling was increased, the expression of *Delta1* and *Hes5* was decreased in the electroporated area of the olfactory pit (Fig. 6A), whereas the expression of *Notch1* was not affected (Fig. 6A). The finding that high BMP activity down-regulates the expression of *Notch1* in explants but not *in vivo,* probably reflects that in intact embryos endogenous signals from nearby tissue stabilize the expression of *Notch1* even in the presence of high BMP activity. Consistently, at stage 22, *Notch1* expression is detected in the entire olfactory epithelium, including the lateral part of the olfactory epithelium (Fig. S3B), known to be exposed to high BMP activity [8].

In contrast, in *Noggin*-transfected embryos (7/10), expression of *Delta1* and *Hes5* was increased, while the expression of *Notch1* was not affected (Fig. 6B). Consistent with previous findings [8], inhibition of BMP activity suppressed the expression of *Id3* and Msx1/2 in the olfactory epithelium, whereas the expression of *Serrate2* was unaffected (4/6) (Fig. S6). Taken together, in olfactory epithelial cells Notch signaling is negatively regulated by BMP activity.

**Figure 7:**
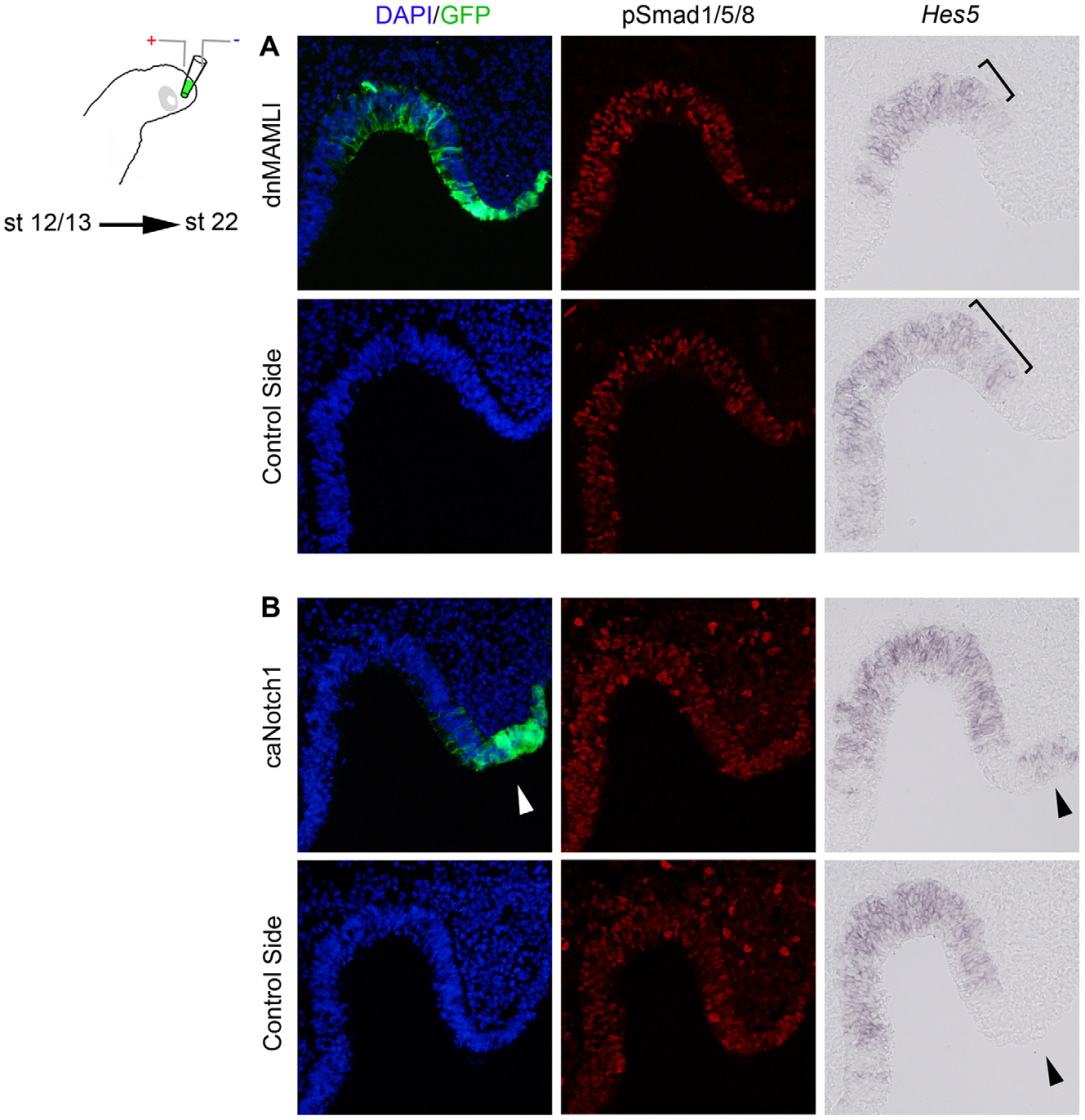
Notch activity does not affect BMP signals in the olfactory epithelium. (**A**, **B**) *In ovo* electroporation of stage 12/13 in the olfactory placodal region using a GFP construct together with a dn*MAMLI* or a ca*Notch1* construct and cultured to stage 22. (**A**) In dn*MAMLI*-electroporated olfactory sensory epithelium, *Hes5* expression was reduced, but pSmad1/5/8 expression was unaffected, compared to the non-electroporated side. (**B**) In ca*Notch1*-electroporated lateral part of the olfactory pit and surface ectoderm, *Hes5* expression was induced, but pSmad1/5/8 expression was unaffected, in the most compared to the non-electroporated side.

Important to note is that modulation of Notch signals did not affect BMP activity *in vitro* or *in vivo*, measured by *Bmp4* and phosphorylated (p) Smad1/5/8 expression (Fig. 7A,B and data not shown). Nevertheless, as expected from previous findings (Fig. S5), in dn*MAMLI*-electroporated embryos, *Hes5* expression was reduced in the olfactory sensory epithelium (7/9) (Fig. 7A). Moreover, in ca*Notch1*-electroporated embryos, *Hes5* expression was induced in the most lateral part of the olfactory pit and surface ectoderm (4/4) (Fig. 7B). In summary, our results provide evidence that BMP signals affect neurogenesis in the olfactory epithelium in part via regulation of Notch activity. This suggests a novel mechanism whereby BMP and Notch signals regulate the generation of early migratory neurons from the olfactory epithelium and the initial formation of the olfactory nerve tract.

## Discussion

The olfactory nerve transmits sensory input from the olfactory epithelium to the olfactory bulb in the telencephalon. The early formation of the olfactory tract has morphologically been well studied [4], [7], [26], but, how external signals regulate the generation of early migrating neurons, and the initial formation of the olfactory nerve have remained elusive. In this study, we provide evidence how migratory neurons and the formation of the olfactory nerve critically depends on a balance of BMP and Notch signals during embryogenesis.

Our results show that all cells that leave the olfactory epithelium exhibit a post-mitotic neuronal character, and that these neurons migrate as a restricted group of cells towards the forebrain, which is in agreement with previous reports in chick [7], [26]. Other studies in rat and mouse have suggested that the epithelioid cells can be of both neuronal and glial character [12], [27], based on the expression of glia markers close to the newly formed olfactory nerve. However, to determine whether the glia cells surrounding the olfactory nerve at later stages are derived from epithelioid cells emanating from the olfactory epithelium, cell lineage analyses have to be conducted.

Our results provide evidence that the generation of migratory neurons is inhibited by increased Notch activity, and that this reduction of migratory neurons is followed by a subsequent failure or complete absence of olfactory nerve formation. This is in agreement with previous suggestions that cells in the migratory mass play a role in building up the olfactory nerve [1], [4], [5]. In contrast, down-regulation of Notch activity does not affect the initial formation of the olfactory nerve, which is characterised by a normal population of migratory neurons compared to generated neurons in the olfactory epithelium. Our results also show that elevated BMP signals suppress the ability of neurons to leave the olfactory epithelium, while inhibition of BMP activity reduces the differentiation of neurons. The decrease of migratory neurons, caused by modulated levels of BMP activity, results in olfactory nerves with reduced thicknesses. Previous studies in endothelial cell migration during vascular development have suggested that BMP signals promote migration [28], whereas Notch activity inhibits migration [29]. In our study, although the olfactory nerves are reduced in thickness, the migration route appears normal, suggesting that BMP and Notch signals are not required for correct spatial migration of migratory neurons derived from the olfactory epithelium or axonal path finding of olfactory sensory neurons towards the olfactory bulb. Rather, the perturbed formation of the olfactory nerve is related to disturbed neurogenesis and the generation of migratory neurons. Detailed knowledge regarding the function of the migratory neurons in correlation with the initial formation of the olfactory nerve remains to be determined.

Not surprisingly, our results show that the regulatory role of Notch signaling in olfactory neurogenesis is to maintain a progenitor pool of cells in the olfactory epithelium. This finding is consistent with other studies, reporting that in the vertebrate peripheral nervous system Notch signals promote proliferation at the expense of differentiation of neuronal cell types [30], [31], [32]. In a recent publication, we have shown that the opposing activities of FGF and BMP signals in the nasal cavity regulate the specification of olfactory sensory and olfactory respiratory epithelial cells, respectively [8]. Moreover, at early olfactory placodal stages, BMP activity inhibits neurogenesis in part by inducing respiratory epithelial cells, but also by suppressing the differentiation of olfactory sensory neurons. The finding that in the absence of BMP activity olfactory sensory cells were arrested in a progenitor state, indicated that BMP activity plays a role in promoting neurogenesis in the olfactory epithelium, but how has remained elusive. In this study our results support and extend this knowledge and provide new insights into this mechanism, showing that at early olfactory placodal stages elevated BMP signals inhibit Notch activity in a dominant manner, which reduce the proliferative pool of cells in the olfactory epithelium. In addition, increased BMP signals suppress neurons ability to leave the olfactory sensory epithelium, thereby negatively regulating the generation of migratory neurons.

On the other hand, inhibition of BMP signals up-regulates Notch activity in the olfactory epithelium, and changes the normal scattered pattern of *Delta1* expression, which indicates that lateral inhibition controls neurogenesis [33], [34], to a uniform *Delta1* expression. Signaling between high *Delta1*-expressing cells has previously been shown to maintain the neural precursor pool during spinal cord development [35]. In addition, the change to a uniform *Hes5* and *Delta1* expression in the absence of BMP signaling resemble the pre-neurogenic expression of these markers during early olfactory placodal stages [6]. Consistently, a functional role for Hes genes in defining the limits of the neurogenic region of the placode has been described [24], suggesting that the initial development of the neurogenic placodal domain might require low levels of BMP signaling prior to lateral inhibition. Taken together, in the olfactory epithelium, up-regulated Notch activity by BMP inhibition maintains cells in a progenitor state and reduces the generation of migratory neurons. A similar role for BMP activity in neurogenesis is observed in the hippocampus of the mouse, where inhibition of BMP activity increases precursor cell populations [36]. In addition, previous results have suggested that BMP signals promote neurogenesis in the chick olfactory sensory epithelium, in mouse olfactory sensory cell lines and in the adult mouse subventricular zone [8], [9], [37]. We now provide a molecular explanation for these observations, that BMP activity is required for the differentiation of neurons by inhibiting Notch activity.

Previous studies concerning neurogenesis in different contexts have reported other interactions between Notch and BMP signaling pathways. In neural crest stem cells, BMP signals are suggested to promote neurogenesis, whereas Notch activity act in a dominant manner over BMP signals and promotes glial differentiation [38]. In addition, in the rhombic lip, Notch signals have been shown to inhibit the responsiveness of progenitor cells to BMP activity, and thereby maintaining them in an undifferentiated state [19]. Our results now provide a novel mechanism during neurogenesis, in which BMP activity negatively regulates Notch signaling in a dominant manner. Since neurogenesis persists into adulthood in the olfactory sensory epithelium, this model system provides information which give a deeper understanding of the molecular interactions that induce stem and precursor cells to differentiate into neurons both at embryonic and adult stages.

In summary, our results provide evidence that a balance of Notch and BMP activity in the olfactory epithelium is critical for neurogenesis and the initial formation of the olfactory nerve. High levels of BMP activity negatively controls neurogenesis in the olfactory epithelium, and the formation of the olfactory nerve, in part via inhibition of Notch activity, while inhibition of BMP signals increase Notch activity and inhibit the differentiation of migratory neurons.

## Materials and Methods

### Embryos

Fertilized white Leghorn chicken (*Gallus gallus*) eggs were obtained from Agrisera AB, Umeå, Sweden. Chick embryos were staged according to the protocol of Hamburger and Hamilton [3]. The use of chick embryos in this study was approved by the Ethical Committee on Animal Experiments for Northern Sweden (Dnr. A26-10).

### *In ovo* electoporation

Stage 12/13 chick embryos were electroporated as previously described [8]. Embryos with GFP expression in the olfactory region were selected for further analysis.

### Olfactory placodal explants

Stage 14 olfactory placodal explants were prepared and cultured as previously described [8]. Human BMP4 (R&D Systems) was used at 35 ng/ml. Noggin conditioned media was used at an estimated concentration of 50 ng/ml. Explants cultured in the presence of control CM generated the same combination of cells as explants cultured alone (data not shown). The γ-secretase inhibitor DAPT (Calbiochem) was used at 50 µM.

### *In situ* hybridization and immunohistochemistry

For the use of *in situ* RNA hybridization and immunohistochemistry, embryos and explants was fixed as described [2], and i*n situ* RNA hybridization was performed essentially as previously described [39]. Chick digoxigenin-labeled probes used were; *Notch1*, *Delta1* [40], *Hes5* (Hes5-1, [41]), *Ngn1* [42], *Id3* [43], *Serrate1* and *Serrate2* [44]. Antibodies used were as follow; anti-HuC/D monoclonal mouse antibody (1∶200) (Molecular Probes), anti-Tuj1 mouse monoclonal antibody (1∶500) (Covenance), anti-a-Caspase3 monoclonal mouse antibody (Cell Signaling, 1∶1000), anti-Msx1/2 monoclonal mouse antibody (4G1, Developmental Studies Hybridoma Bank, 1∶20), rabbit anti-pSmad1/5/8 (Cell Signaling 1∶800), rabbit anti-p-Histone3 (Millipore, 1∶500) and anti-GFP rabbit antibody (1∶300) (Molecular probes). Nuclei were stained using DAPI (Sigma).

### Tissue preparation for 3D analysis

Tissue preparation of chick heads for OPT scanning was essentially performed as previously described [45] with the following changes; bleaching with H_2_O_2_:DMSO:MeOH was not performed until after antibody incubations due to epitope sensitivity, tissue was not repeatedly brought to −80°C and room temperature.

### Optical projection tomography

OPT scanning, using the Bioptonics 3001 OPT scanner (Bioptonics) visualizing goat anti-rabbit Alexa 488 (1∶400) (Molecular Probes) and goat anti-mouse Alexa 594 (1∶400) (Molecular Probes) respectively, was performed as previously described [45].

### Statistical analysis

HuC/D positive cells in dn*MAMLI*, ca*Notch1*, *Alk6*, *Noggin* and control *GFP* electroporated embryos were quantified and compared with the analogous HuC/D positive cells on the non-electroporated control side. The total amount of cells was determined by counting the number of nuclei using DAPI (Boehringer Mannheim). The graphs represent mean number ± SEM as a percentage of total cell number. Significance (*) was determined by Student's t-Test p<0.05.

## Supporting Information

Movie S1 Three-dimensional reconstruction and rotation of the head of a control-GFP vector electroporated embryo stained for Tuj1. The olfactory nerve on the electroporated right side has formed normally and extends all the way to the telencephalon.
(MOV)

Movie S2 Three-dimensional reconstruction and rotation of the head of a caNotch1 vector electroporated embryo on the right side of the olfactory epithelial region and stained for Tuj1. The olfactory nerve has completely failed to form on the electroporated right side of the embryo.
(MOV)

Figure S1 Modulated Notch and BMP activity does not affect cell death in the olfactory epithelium. Analysis of activated (a) Caspase3 expression (red) in the olfactory pit region after *in ovo* electroporation of stage 12/13 chick embryos in the olfactory placodal region using a GFP construct (green) alone (n = 8) or together with *Alk6* (n = 3), *Noggin* (n = 5) and ca*Notch1* (n = 5), and cultured to approximately stage 19. None of the electroporated constructs resulted in any significant changes in the number of aCaspase3 positive cells compared with the non-elctroporated control side. p<0,05 is considered significant by using Student's t-Test.
(TIF)

Figure S2 Modulations in BMP and Notch activity change the number of neurons in the olfactory epithelium and the migratory mass. Stage 12/13 chick embryos were electroporated *in ovo* in the olfactory placodal region using *GFP* (n = 6) alone or together with *Alk6* (n = 7), *Noggin* (n = 7), ca*Notch1* (n = 7) or dn*MAMLI* (n = 7) and cultured to approximately stage 19. (**A-E**) The total numbers of non-electroporated HuC/D^+^ (red) neurons and electroporated HuC/D^+^/GFP^+^ (yellow) neurons in the olfactory epithelium (OE) and the migratory mass (MM) was quantified on both the electroporated side and the control side. Error bars are based on the total number of neurons.
(TIF)

Figure S3 Expression pattern of *Notch1*, *Delta1*, *Serrate1* and *Serrate2* in the olfactory epithelium. Schematic drawings of stage 17 and 22 chick embryos to the left indicate the position of the transversal sections of the olfactory pit shown in the following panels. (**A**) At stage 17, *Notch1* and *Delta1* are expressed in a majority of cells in the olfactory placode, but no expression of *Serrate1* and *Serrate2* can be detected. (**B**) At stage 22, *Notch1* expression is detected in the apical part throughout the olfactory pit and *Delta1* is expressed in the medial part of the olfactory pit in a salt-and-pepper pattern. *Serrate1* and *Serrate2* are expressed weaker compared to *Delta1* in the medial part of the olfactory pit.
(TIF)

Figure S4 Inhibition of Notch activity results in decreased proliferation and increased differentiation of epithelioid cells. Analysis of pHistone3 expression (red) in the olfactory pit region after *in ovo* electroporation of stage 12/13 embryos in the olfactory placodal region using a GFP construct alone (n =  8) or together with a dn*MAMLI* construct (n = 6) and cultured to stage 19. A significant decrease in cell proliferation was detected in dn*MAMLI*-electroporated embryos compared to control embryos. p<0,05 is considered significant by using Student's t-Test.
(TIF)

Figure S5 Notch activity is required to maintain *Hes5* positive progenitor cells *in vivo*. *In ovo* electroporation of stage 12/13 in the olfactory placodal region using a GFP construct together with a dn*MAMLI* construct and cultured to stage 19. A decrease in *Hes5* expression and an increase in the generation of *Ngn1* ^+^ and HuC/D^+^ cells was detected in dn*MAMLI*-electroporated part of the olfactory epithelium compared to the non-electroporated side.
(TIF)

Figure S6 Inhibition of BMP activity reduces *Id3* and Msx1/2, but not *Serrate2* expression. *In ovo* electroporation of stage 12/13 in the olfactory placodal region using a GFP construct together with a *Noggin* construct and cultured to stage 22. Inhibition of BMP activity suppressed the expression of *Id3* and Msx1/2 in the olfactory epithelium, whereas the expression of *Serrate2* was unaffected.
(TIF)

Methods S1 (DOC)

## References

[1] Croucher SJ, Tickle C (1989). Characterization of epithelial domains in the nasal passages of chick embryos: spatial and temporal mapping of a range of extracellular matrix and cell surface molecules during development of the nasal placode.. Development.

[2] Sjodal M, Edlund T, Gunhaga L (2007). Time of exposure to BMP signals plays a key role in the specification of the olfactory and lens placodes ex vivo.. Dev Cell.

[3] Hamburger V, Hamilton HL (1992). A series of normal stages in the development of the chick embryo. 1951.. Dev Dyn.

[4] Drapkin PT, Silverman AJ (1999). Development of the chick olfactory nerve.. Dev Dyn.

[5] Fornaro M, Geuna S, Fasolo A, Giacobini-Robecchi MG (2003). HuC/D confocal imaging points to olfactory migratory cells as the first cell population that expresses a post-mitotic neuronal phenotype in the chick embryo.. Neuroscience.

[6] Maier E, Gunhaga L (2009). Dynamic expression of neurogenic markers in the developing chick olfactory epithelium.. Dev Dyn.

[7] Mendoza AS, Breipohl W, Miragall F (1982). Cell migration from the chick olfactory placode: a light and electron microscopic study.. J Embryol Exp Morphol.

[8] Maier E, von Hofsten J, Nord H, Fernandes M, Paek H (2010). Opposing Fgf and Bmp activities regulate the specification of olfactory sensory and respiratory epithelial cell fates.. Development.

[9] Shou J, Murray RC, Rim PC, Calof AL (2000). Opposing effects of bone morphogenetic proteins on neuron production and survival in the olfactory receptor neuron lineage.. Development.

[10] Shou J, Rim PC, Calof AL (1999). BMPs inhibit neurogenesis by a mechanism involving degradation of a transcription factor.. Nat Neurosci.

[11] Orita Y, Nagatsuka H, Tsujigiwa H, Yoshinobu J, Maeda Y (2006). Expression of Notch1 and Hes5 in the developing olfactory epithelium.. Acta Otolaryngol.

[12] Schwarting GA, Gridley T, Henion TR (2007). Notch1 expression and ligand interactions in progenitor cells of the mouse olfactory epithelium.. J Mol Histol.

[13] Yoon K, Gaiano N (2005). Notch signaling in the mammalian central nervous system: insights from mouse mutants.. Nat Neurosci.

[14] Bray SJ (2006). Notch signalling: a simple pathway becomes complex.. Nat Rev Mol Cell Biol.

[15] Iso T, Kedes L, Hamamori Y (2003). HES and HERP families: multiple effectors of the Notch signaling pathway.. J Cell Physiol.

[16] Basak O, Taylor V (2007). Identification of self-replicating multipotent progenitors in the embryonic nervous system by high Notch activity and Hes5 expression.. Eur J Neurosci.

[17] Ohtsuka T, Ishibashi M, Gradwohl G, Nakanishi S, Guillemot F (1999). Hes1 and Hes5 as notch effectors in mammalian neuronal differentiation.. Embo J.

[18] Maillard I, Weng AP, Carpenter AC, Rodriguez CG, Sai H (2004). Mastermind critically regulates Notch-mediated lymphoid cell fate decisions.. Blood.

[19] Machold RP, Kittell DJ, Fishell GJ (2007). Antagonism between Notch and bone morphogenetic protein receptor signaling regulates neurogenesis in the cerebellar rhombic lip.. Neural Dev.

[20] Timmer JR, Wang C, Niswander L (2002). BMP signaling patterns the dorsal and intermediate neural tube via regulation of homeobox and helix-loop-helix transcription factors.. Development.

[21] James RG, Schultheiss TM (2005). Bmp signaling promotes intermediate mesoderm gene expression in a dose-dependent, cell-autonomous and translation-dependent manner.. Dev Biol.

[22] Geisert EE, Frankfurter A (1989). The neuronal response to injury as visualized by immunostaining of class III beta-tubulin in the rat.. Neurosci Lett.

[23] Cau E, Casarosa S, Guillemot F (2002). Mash1 and Ngn1 control distinct steps of determination and differentiation in the olfactory sensory neuron lineage.. Development.

[24] Cau E, Gradwohl G, Casarosa S, Kageyama R, Guillemot F (2000). Hes genes regulate sequential stages of neurogenesis in the olfactory epithelium.. Development.

[25] Tian G, Ghanekar SV, Aharony D, Shenvi AB, Jacobs RT (2003). The mechanism of gamma-secretase: multiple inhibitor binding sites for transition state analogs and small molecule inhibitors.. J Biol Chem.

[26] Fornaro M, Geuna S, Fasolo A, Giacobini-Robecchi MG (2001). Evidence of very early neuronal migration from the olfactory placode of the chick embryo.. Neuroscience.

[27] Valverde F, Santacana M, Heredia M (1992). Formation of an olfactory glomerulus: morphological aspects of development and organization.. Neuroscience.

[28] Valdimarsdottir G, Goumans MJ, Rosendahl A, Brugman M, Itoh S (2002). Stimulation of Id1 expression by bone morphogenetic protein is sufficient and necessary for bone morphogenetic protein-induced activation of endothelial cells.. Circulation.

[29] Itoh F, Itoh S, Goumans MJ, Valdimarsdottir G, Iso T (2004). Synergy and antagonism between Notch and BMP receptor signaling pathways in endothelial cells.. Embo J.

[30] Lassiter RN, Ball MK, Adams JS, Wright BT, Stark MR (2010). Sensory neuron differentiation is regulated by notch signaling in the trigeminal placode.. Dev Biol.

[31] Tang LS, Alger HM, Pereira FA (2006). COUP-TFI controls Notch regulation of hair cell and support cell differentiation.. Development.

[32] Tsarovina K, Schellenberger J, Schneider C, Rohrer H (2008). Progenitor cell maintenance and neurogenesis in sympathetic ganglia involves Notch signaling.. Mol Cell Neurosci.

[33] Haddon C, Smithers L, Schneider-Maunoury S, Coche T, Henrique D (1998). Multiple delta genes and lateral inhibition in zebrafish primary neurogenesis.. Development.

[34] le Roux I, Lewis J, Ish-Horowicz D (2003). Notch activity is required to maintain floorplate identity and to control neurogenesis in the chick hindbrain and spinal cord.. Int J Dev Biol.

[35] Akai J, Halley PA, Storey KG (2005). FGF-dependent Notch signaling maintains the spinal cord stem zone.. Genes Dev.

[36] Bonaguidi MA, Peng CY, McGuire T, Falciglia G, Gobeske KT (2008). Noggin expands neural stem cells in the adult hippocampus.. J Neurosci.

[37] Colak D, Mori T, Brill MS, Pfeifer A, Falk S (2008). Adult neurogenesis requires Smad4-mediated bone morphogenic protein signaling in stem cells.. J Neurosci.

[38] Morrison SJ, Perez SE, Qiao Z, Verdi JM, Hicks C (2000). Transient Notch activation initiates an irreversible switch from neurogenesis to gliogenesis by neural crest stem cells.. Cell.

[39] Wilkinson DG, Nieto MA (1993). Detection of messenger RNA by in situ hybridization to tissue sections and whole mounts.. Methods Enzymol.

[40] Vargesson N, Patel K, Lewis J, Tickle C (1998). Expression patterns of Notch1, Serrate1, Serrate2 and Delta1 in tissues of the developing chick limb.. Mech Dev.

[41] Fior R, Henrique D (2005). A novel hes5/hes6 circuitry of negative regulation controls Notch activity during neurogenesis.. Dev Biol.

[42] Perez SE, Rebelo S, Anderson DJ (1999). Early specification of sensory neuron fate revealed by expression and function of neurogenins in the chick embryo.. Development.

[43] Kee Y, Bronner-Fraser M (2001). The transcriptional regulator Id3 is expressed in cranial sensory placodes during early avian embryonic development.. Mech Dev.

[44] Laufer E, Dahn R, Orozco OE, Yeo CY, Pisenti J (1997). Expression of Radical fringe in limb-bud ectoderm regulates apical ectodermal ridge formation.. Nature.

[45] Alanentalo T, Loren CE, Larefalk A, Sharpe J, Holmberg D (2008). High-resolution three-dimensional imaging of islet-infiltrate interactions based on optical projection tomography assessments of the intact adult mouse pancreas.. J Biomed Opt.

