# Supplementary figures and images for: A Balance of BMP and Notch Activity Regulates Neurogenesis and Olfactory Nerve Formation
Source: PLoS One. 2011 Feb 23;6(2):e17379. doi: 10.1371/journal.pone.0017379 (PMC3044177; doi:10.1371/journal.pone.0017379)

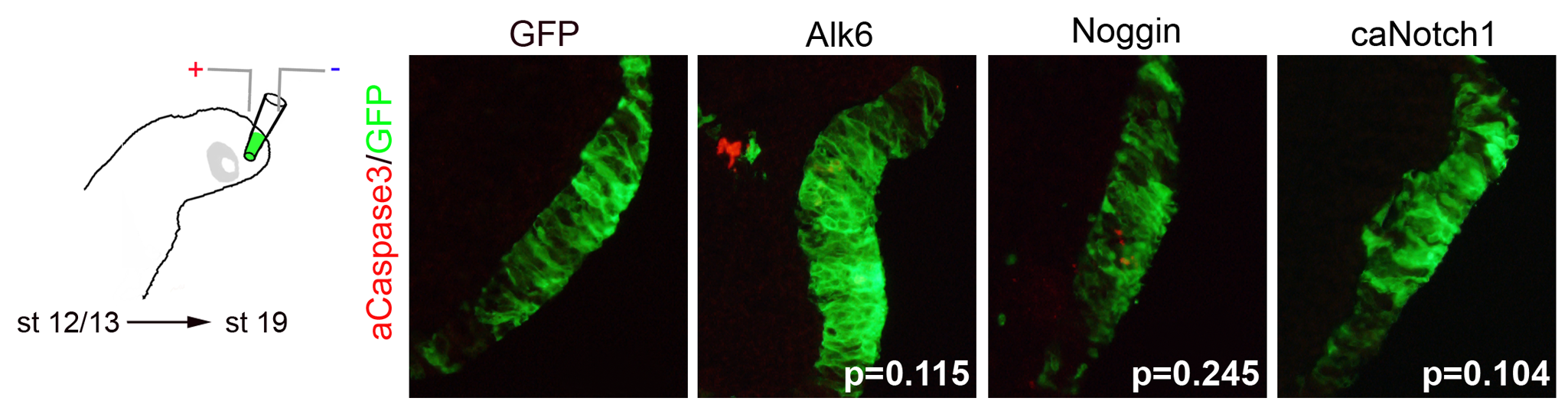

Supplement: Figure S1 — Modulated Notch and BMP activity does not affect cell death in the olfactory epithelium. Analysis of activated (a) Caspase3 expression (red) in the olfactory pit region after in ovo electroporation of stage 12/13 chick embryos in the olfactory placodal region using a GFP construct (green) alone (n = 8) or together with Alk6 (n = 3), Noggin (n = 5) and caNotch1 (n = 5), and cultured to approximately stage 19. None of the electroporated constructs resulted in any significant changes in the number of aCaspase3 positive cells compared with the non-elctroporated control side. p<0,05 is considered significant by using Student's t-Test. (TIF) [file pone.0017379.s003.tif]

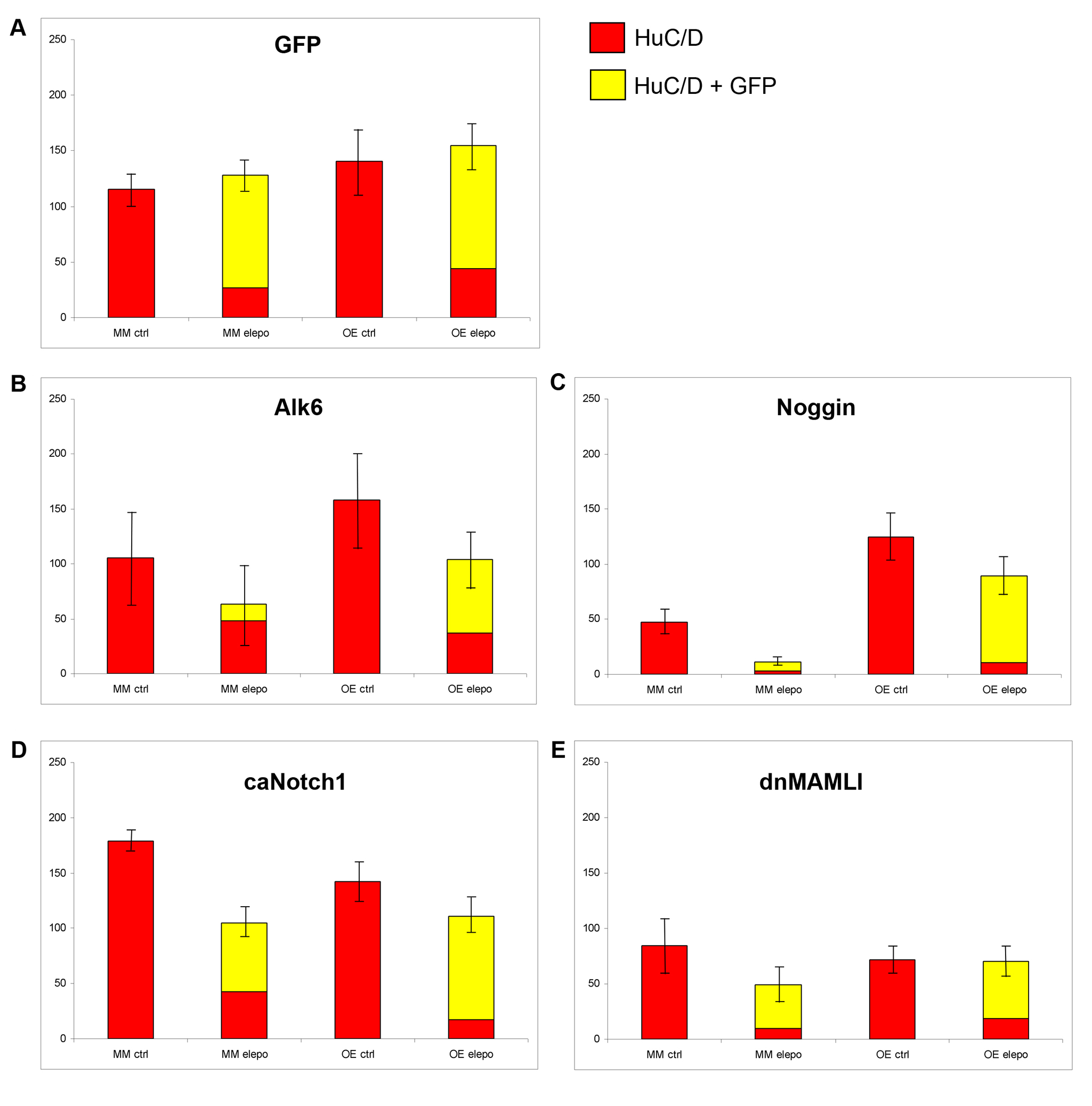

Supplement: Figure S2 — Modulations in BMP and Notch activity change the number of neurons in the olfactory epithelium and the migratory mass. Stage 12/13 chick embryos were electroporated in ovo in the olfactory placodal region using GFP (n = 6) alone or together with Alk6 (n = 7), Noggin (n = 7), caNotch1 (n = 7) or dnMAMLI (n = 7) and cultured to approximately stage 19. (A-E) The total numbers of non-electroporated HuC/D+ (red) neurons and electroporated HuC/D+/GFP+ (yellow) neurons in the olfactory epithelium (OE) and the migratory mass (MM) was quantified on both the electroporated side and the control side. Error bars are based on the total number of neurons. (TIF) [file pone.0017379.s004.tif]

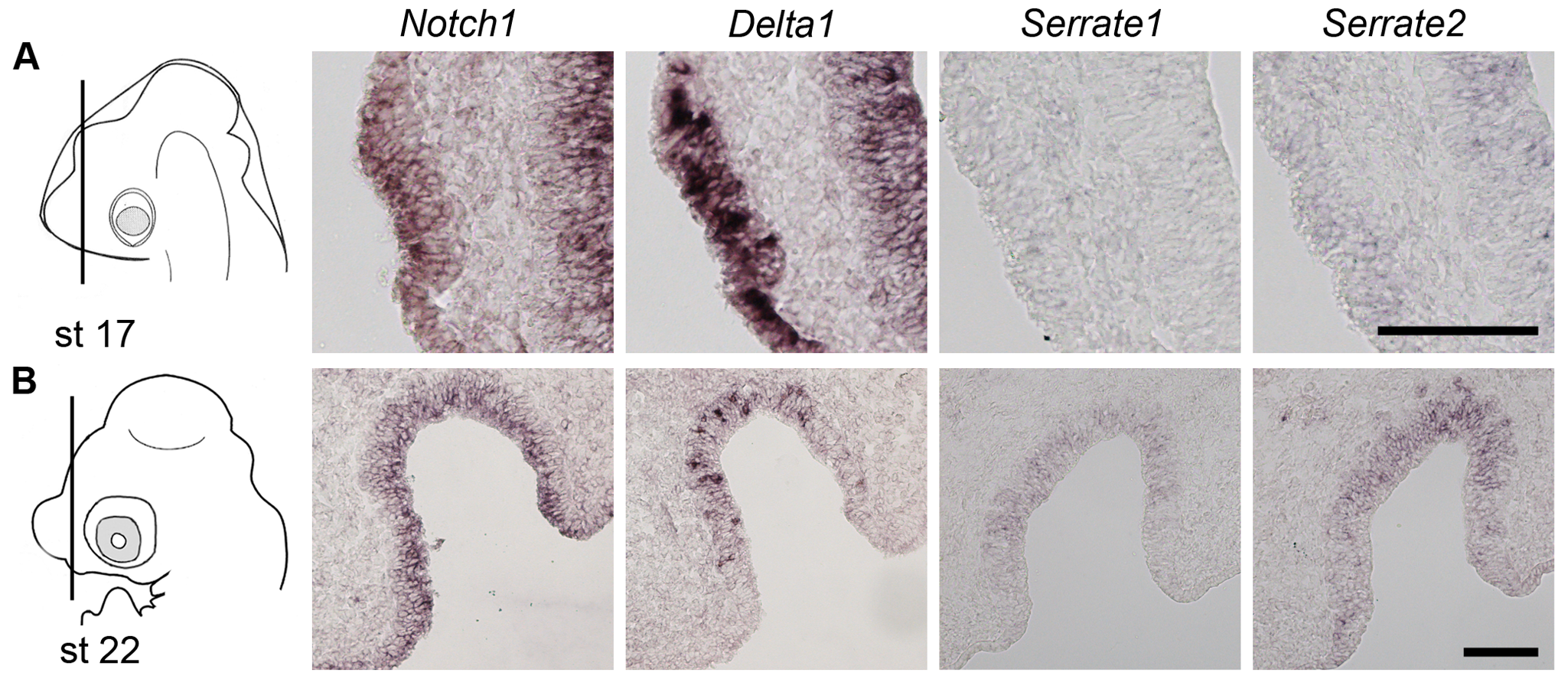

Supplement: Figure S3 — Expression pattern of Notch1, Delta1, Serrate1 and Serrate2 in the olfactory epithelium. Schematic drawings of stage 17 and 22 chick embryos to the left indicate the position of the transversal sections of the olfactory pit shown in the following panels. (A) At stage 17, Notch1 and Delta1 are expressed in a majority of cells in the olfactory placode, but no expression of Serrate1 and Serrate2 can be detected. (B) At stage 22, Notch1 expression is detected in the apical part throughout the olfactory pit and Delta1 is expressed in the medial part of the olfactory pit in a salt-and-pepper pattern. Serrate1 and Serrate2 are expressed weaker compared to Delta1 in the medial part of the olfactory pit. (TIF) [file pone.0017379.s005.tif]

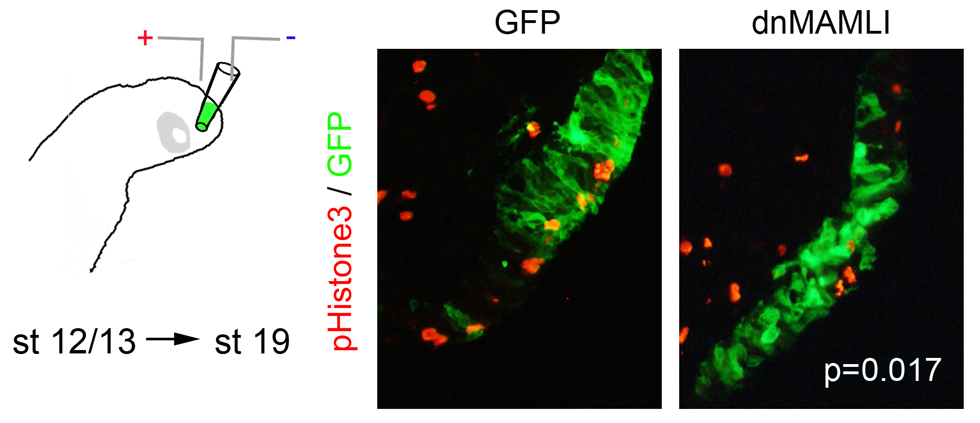

Supplement: Figure S4 — Inhibition of Notch activity results in decreased proliferation and increased differentiation of epithelioid cells. Analysis of pHistone3 expression (red) in the olfactory pit region after in ovo electroporation of stage 12/13 embryos in the olfactory placodal region using a GFP construct alone (n = 8) or together with a dnMAMLI construct (n = 6) and cultured to stage 19. A significant decrease in cell proliferation was detected in dnMAMLI-electroporated embryos compared to control embryos. p<0,05 is considered significant by using Student's t-Test. (TIF) [file pone.0017379.s006.tif]

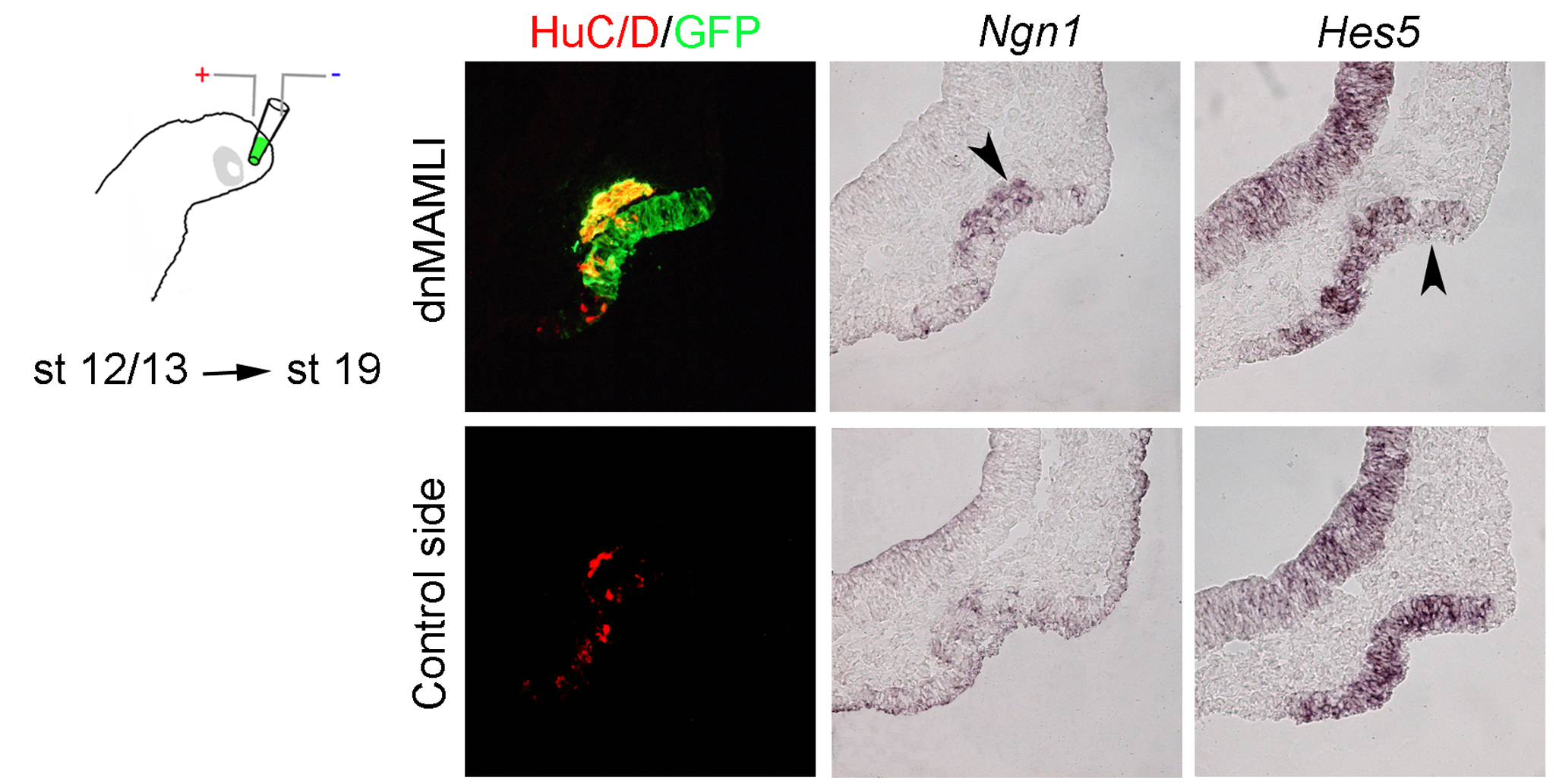

Supplement: Figure S5 — Notch activity is required to maintain Hes5 positive progenitor cells in vivo. In ovo electroporation of stage 12/13 in the olfactory placodal region using a GFP construct together with a dnMAMLI construct and cultured to stage 19. A decrease in Hes5 expression and an increase in the generation of Ngn1 + and HuC/D+ cells was detected in dnMAMLI-electroporated part of the olfactory epithelium compared to the non-electroporated side. (TIF) [file pone.0017379.s007.tif]

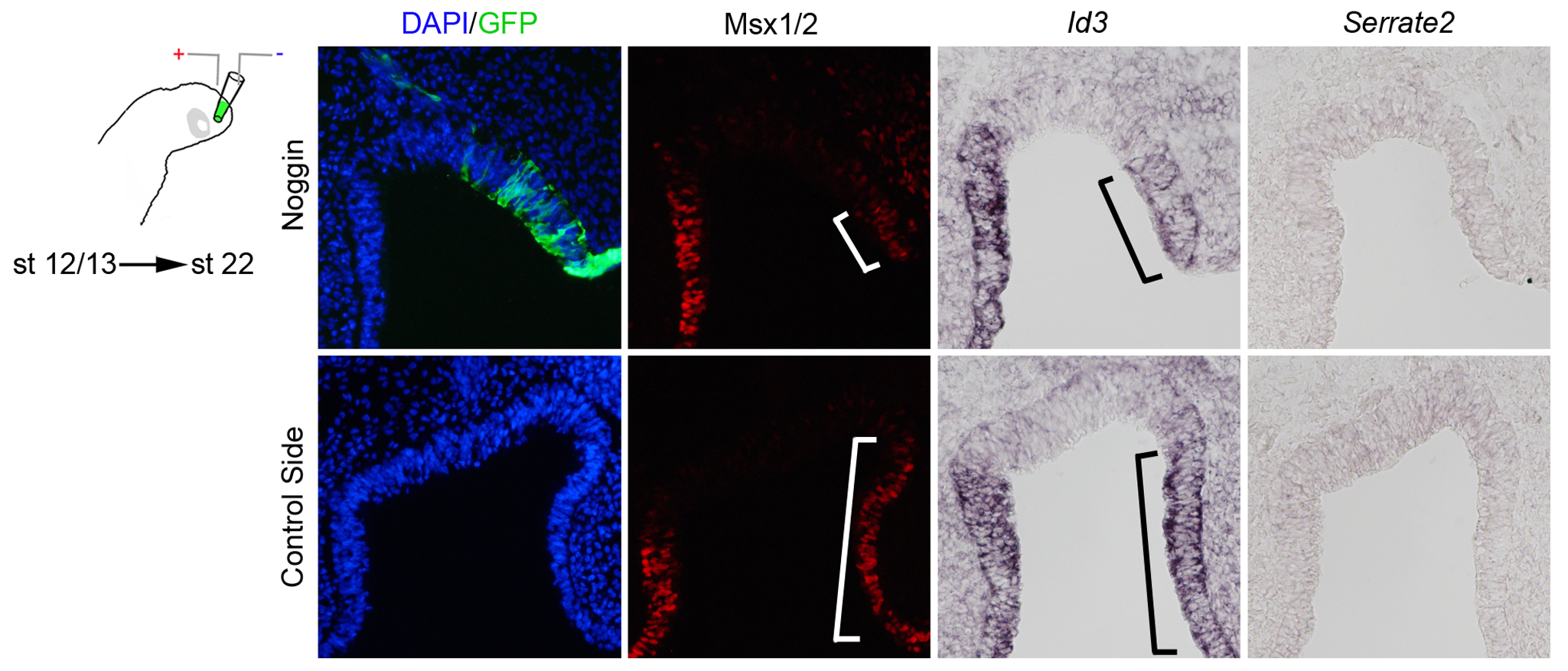

Supplement: Figure S6 — Inhibition of BMP activity reduces Id3 and Msx1/2, but not Serrate2 expression. In ovo electroporation of stage 12/13 in the olfactory placodal region using a GFP construct together with a Noggin construct and cultured to stage 22. Inhibition of BMP activity suppressed the expression of Id3 and Msx1/2 in the olfactory epithelium, whereas the expression of Serrate2 was unaffected. (TIF) [file pone.0017379.s008.tif]
