# Supplementary material for: A Balance of BMP and Notch Activity Regulates Neurogenesis and Olfactory Nerve Formation
Source: PLoS One. 2011 Feb 23;6(2):e17379. doi: 10.1371/journal.pone.0017379 (PMC3044177; doi:10.1371/journal.pone.0017379)
Supplement: Methods S1 — (DOC) [file pone.0017379.s009.doc]

**Supporting Materials and Methods**

Statistical analysis

Active Caspase 3 (aCaspase3) positive cells in Alk6, Noggin, caNotch1 and control GFP electroporated embryos, and phosphorylated Histone 3 (pHistone3) in dnMAMLI and GFP-electroporated embryos, were quantified and compared with the total number of cells, determined by DAPI stained nuclei (Boehringer Mannheim). The graphs represent mean number ± SEM as a percentage of total cell number. *P*-values were obtained comparing GFP-control with GFP-construct-electroporated embryos. Significance (*) was determined by Student’s t-Test p < 0.05.
